# Supplementary material for: Cross-Frequency Slow Oscillation–Spindle Coupling in a Biophysically Realistic Thalamocortical Neural Mass Model
Source: Front Comput Neurosci. 2022 May 6;16:769860. doi: 10.3389/fncom.2022.769860 (PMC9120371; doi:10.3389/fncom.2022.769860)
Supplement: Supplementary file 1 [file Data_Sheet_1.pdf]

# Supplementary Material for "Cross-Frequency Slow Oscillation–Spindle Coupling in a Biophysically Realistic Thalamocortical Neural Mass Model"

## S1 PRECOMPUTED TRANSFER FUNCTIONS OF THE CORTICAL MODEL

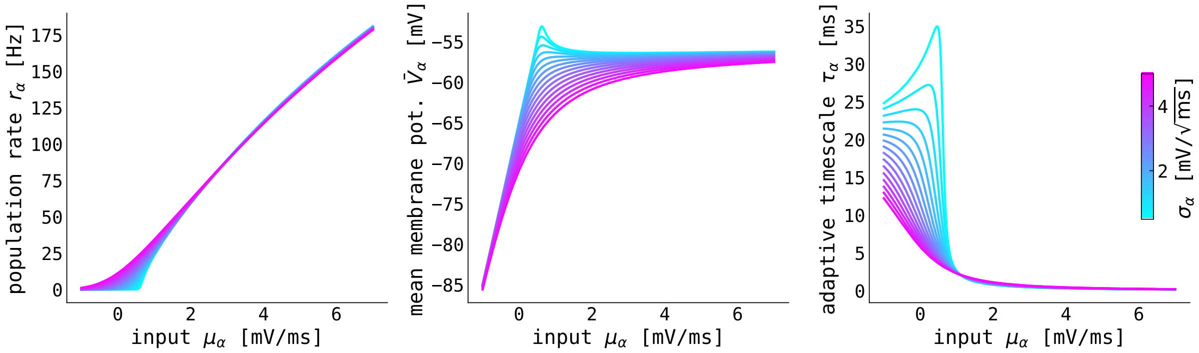


**Figure S1. Precomputed quantities of the linear-nonlinear cascade model.** Panels show (left to right) the nonlinear transfer functions $\Phi_r$ for the population firing rate $r_\alpha$ (cf. Eq. 16), $\Phi_V$ for the mean membrane voltage $\bar{V}_\alpha$ (cf. Eq. 17), and $\Phi_\tau$ for the adaptive timescale $\tau_\alpha$ (cf. Eq. 18). The color coding represents the level of input current variance $\sigma_\alpha$ across the population. The linear-nonlinear cascade was precomputed with the following single AdEx neuron parameters (see Eqs. (11) and (12)): membrane capacitance $C_{m,c} = 200$ pF, leak conductance $g_L = 10$ nS, membrane time constant $\tau_m = C/g_L = 20$ ms, leak reversal potential $E_L = -65$ mV, threshold slope factor $\Delta_T = 1.5$ mV, spike initiation voltage threshold $V_T = -50$ mV, spike voltage threshold $V_s = -40$ mV, reset voltage $V_r = -70$ mV, refractory period $T_{ref} = 1.5$ ms. Note, that the somatic adaptation current (Eq. (13)) is not included in the linear-nonlinear cascade precomputation.

## S2 CORTICAL MODEL WITH FINITE NOISE DRIVEN BY THALAMIC INPUT

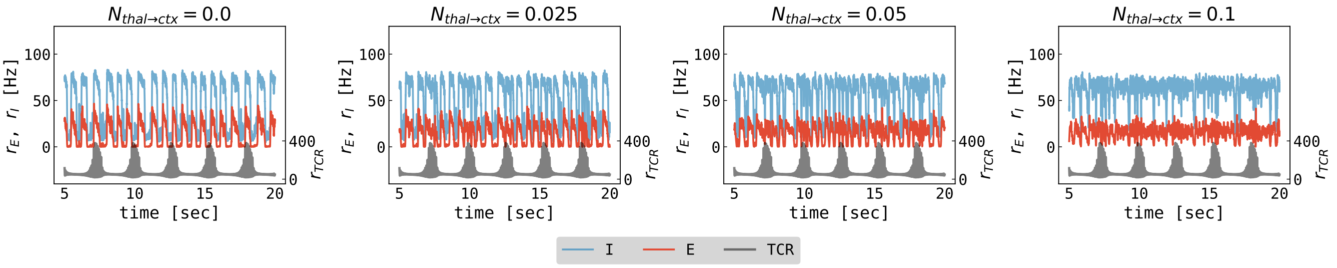


**Figure S2. Cortical response to thalamic spindle stimulation with cortical noise.** The panels show the firing rates of the excitatory cortical population (red), the inhibitory cortical population (blue), and the thalamic input (black) as function of time. The baseline in DOWN states for excitatory (inhibitory) population is around 0 Hz (50 Hz). Cortical model is parametrized at the border of the limit cycle and the UP state ($\mu_E = 0.65$ nA). Different columns show distinct values of thalamus $\to$ cortex connection strength ranging from $N_{thal\to ctx} = 0.0$ to $N_{thal\to ctx} = 0.1$. The parameters for the cortical node were: $\mu_I = 0.4$ nA, $\sigma_E = \sigma_I = 0.05$ $\mathrm{mV/ms^{3/2}}$, for all other parameters see Table S2. The parameters for thalamic node were: $g_{LK} = 0.031$ $\mathrm{mS/cm^2}$, $g_h = 0.062$ $\mathrm{mS/cm^2}$, $\sigma_{TCR} = 0.0$ $\mathrm{mV/ms^{3/2}}$, for all other parameters see Table S1. The parameters of connected thalamocortical model are given in Table S3.

## S3 SLOW OSCILLATION–SPINDLE INTERACTION IN THE UP STATE REGIME WITH LONG UP STATES

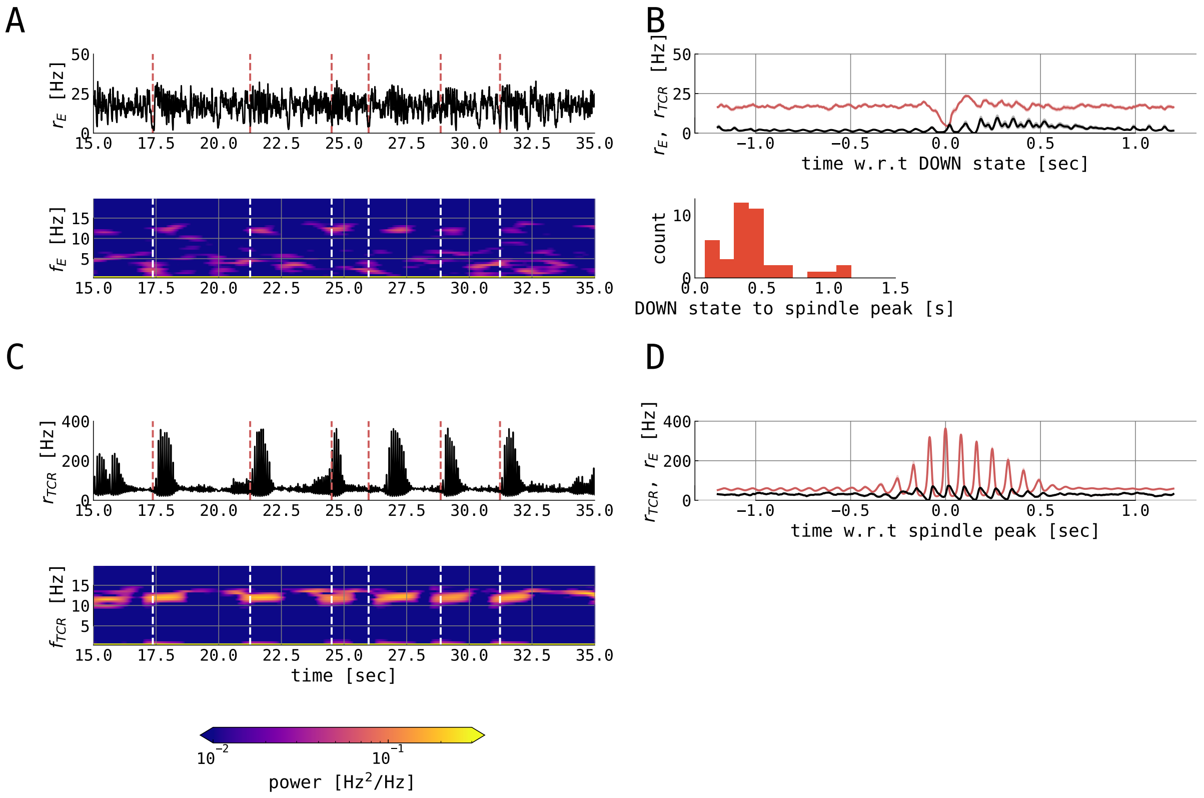


**Figure S3. Spindles in the thalamocortical motif with long UP states.** The figure shows various variables from 120 second of simulation of full thalamocortical model simulation with the cortical node being parametrized at the right border between the slow limit cycle and the UP state, further away from the bifurcation line ($\mu_E = 0.66$ nA). Individual panels show: (**A**) 20 seconds time series excerpt of the firing rate of the excitatory population in the cortical node (black) and its slow oscillation phase (blue) computed using the Hilbert transform of low-pass filtered cortical excitatory firing rate. The panel below shows the time-frequency representation of the cortical excitatory firing rates computed using the Short Time Fourier Transform with a 2 second time window. Dashed vertical lines (red in time series plot and white in time-frequency plot) denote the midpoints of cortical DOWN states which are followed by a cortical spindle in the 1.5 second window. (**B**) the mean $\pm$ the SEM of the cortical excitatory firing rates (red) and TCR firing rates (black), locked on the cortical DOWN states for the whole interval of 120 seconds. (**C**) 20 seconds time series excerpt of the firing rate of the thalamocortical relay population in the thalamic node (black). The panel below shows the time-frequency representation of the TCR firing rates computed using the Short Time Fourier Transform with a 2 second time window. Dashed vertical lines (red in time series plot and white in time-frequency plot) denote the midpoints of cortical DOWN states which are followed by a cortical spindle in the 1.5 second window. (**D**) the mean $\pm$ the SEM of the TCR firing rates (red) and cortical excitatory firing rates (black), locked on the thalamic spindle peaks in the whole interval of 120 seconds. The panel below shows the distribution of delays between midpoints of cortical DOWN states and spindle-band peaks. Note, that the the long UP states hinder slow oscillation phase estimation, since in a particularly long UP state, the Hilbert phase resets and no longer maps onto DOWN states with $\phi_{ctx,SO} = -\pi = \pi$ and UP states with $\phi_{ctx,SO} = 0$. For this reason we do not quantify the cortical slow oscillation phase. The model was simulated with $N_{thal \to ctx} = 0.12$, $N_{ctx \to thal} = 1.2$, $\mu_I = 0.4$ nA, $\sigma_E = \sigma_I = 0.05\ \mathrm{mV/ms^{3/2}}$, $\sigma_{TCR} = 0.005\ \mathrm{mV/ms^{3/2}}$, while other parameters were kept constant as per tables S1, S2, and S3.

## S4 SLOW OSCILLATION–SPINDLE INTERACTION IN THE DOWN STATE REGIME

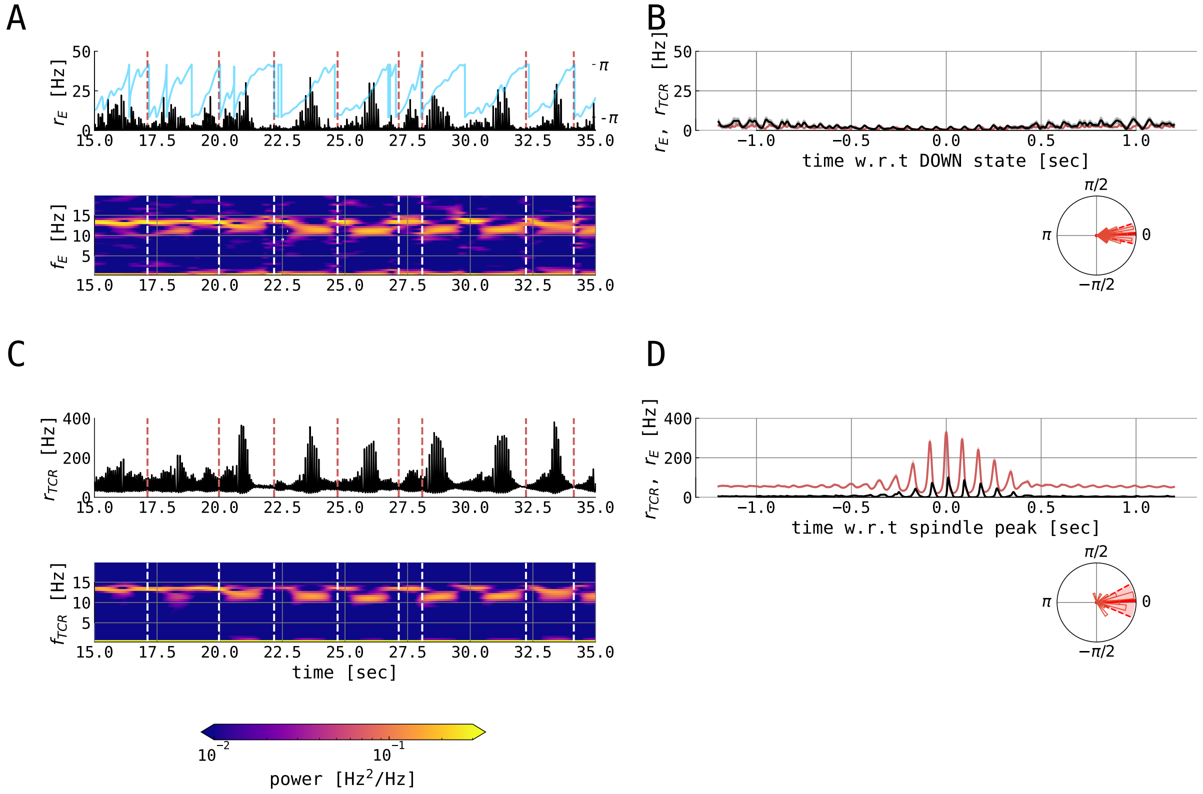


**Figure S4. Spindles in the thalamocortical motif with the DOWN state parametrization.** The figure shows various variables from 120 second of simulation of full thalamocortical model simulation with the cortical node being parametrized at the left border between the slow limit cycle and the DOWN state ($\mu_E = 0.36$ nA). Individual panels show: (**A**) 20 seconds time series excerpt of the firing rate of the excitatory population in the cortical node (black) and its slow oscillation phase (blue) computed using the Hilbert transform of low-pass filtered cortical excitatory firing rate. The panel below shows the time-frequency representation of the cortical excitatory firing rates computed using the Short Time Fourier Transform with a 2 second time window. Dashed vertical lines (red in time series plot and white in time-frequency plot) denote the midpoints of cortical DOWN states which are followed by a cortical spindle in the 1.5 second window. (**B**) the mean $\pm$ the SEM of the cortical excitatory firing rates (red) and TCR firing rates (black), locked on the cortical DOWN states for the whole interval of 120 seconds. (**C**) 20 seconds time series excerpt of the firing rate of the thalamocortical relay population in the thalamic node (black). The panel below shows the time-frequency representation of the TCR firing rates computed using the Short Time Fourier Transform with a 2 second time window. Dashed vertical lines (red in time series plot and white in time-frequency plot) denote the midpoints of cortical DOWN states which are followed by a cortical spindle in the 1.5 second window. (**D**) the mean $\pm$ the SEM of the TCR firing rates (red) and cortical excitatory firing rates (black), locked on the thalamic spindle peaks in the whole interval of 120 seconds. The panel below shows the distribution of cortical slow oscillation phases for the maximum of the thalamic fast spindle band peak. Shown are histogram bars in thin red and circular mean ($\pm$ circular STD) in thick (dashed) red. The model was simulated with $N_{thal \to ctx} = 0.12$, $N_{ctx \to thal} = 1.2$, $\mu_I = 0.4$ nA, $\sigma_E = \sigma_I = 0.05\ \mathrm{mV/ms^{3/2}}$, $\sigma_{TCR} = 0.005\ \mathrm{mV/ms^{3/2}}$, while other parameters were kept constant as per tables S1, S2, and S3.

# S5 SLOW OSCILLATION–SPINDLE INTERACTION IN THE LIMIT CYCLE REGIME

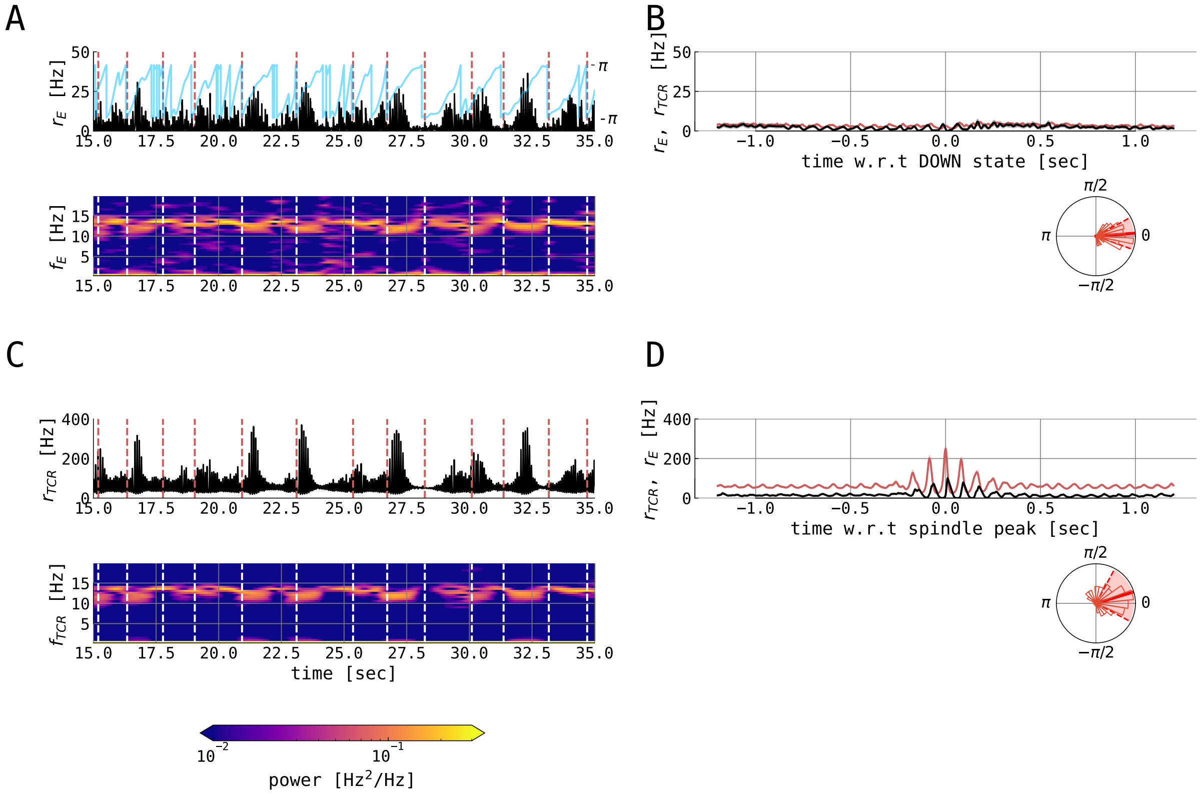


**Figure S5. Spindles in the thalamocortical motif with the limit cycle parametrization.** The figure shows various variables from 120 second of simulation of full thalamocortical model simulation with the cortical node being parametrized inside the slow limit cycle ($\mu_E = 0.42$ nA). Individual panels show: (**A**) 20 seconds time series excerpt of the firing rate of the excitatory population in the cortical node (black) and its slow oscillation phase (blue) computed using the Hilbert transform of low-pass filtered cortical excitatory firing rate. The panel below shows the time-frequency representation of the cortical excitatory firing rates computed using the Short Time Fourier Transform with a 2 second time window. Dashed vertical lines (red in time series plot and white in time-frequency plot) denote the midpoints of cortical DOWN states which are followed by a cortical spindle in the 1.5 second window. (**B**) the mean $\pm$ the SEM of the cortical excitatory firing rates (red) and TCR firing rates (black), locked on the cortical DOWN states for the whole interval of 120 seconds. (**C**) 20 seconds time series excerpt of the firing rate of the thalamocortical relay population in the thalamic node (black). The panel below shows the time-frequency representation of the TCR firing rates computed using the Short Time Fourier Transform with a 2 second time window. Dashed vertical lines (red in time series plot and white in time-frequency plot) denote the midpoints of cortical DOWN states which are followed by a cortical spindle in the 1.5 second window. (**D**) the mean $\pm$ the SEM of the TCR firing rates (red) and cortical excitatory firing rates (black), locked on the thalamic spindle peaks in the whole interval of 120 seconds. The panel below shows the distribution of cortical slow oscillation phases for the maximum of the thalamic fast spindle band peak. Shown are histogram bars in thin red and circular mean ($\pm$ circular STD) in thick (dashed) red. The model was simulated with $N_{thal \to ctx} = 0.12$, $N_{ctx \to thal} = 1.2$, $\mu_I = 0.4$ nA, $\sigma_E = \sigma_I = 0.05$ mV/ms$^{3/2}$, $\sigma_{TCR} = 0.005$ mV/ms$^{3/2}$, while other parameters were kept constant as per tables S1, S2, and S3.

## S6 FULL SET OF MODEL EQUATIONS FOR THE THALAMIC MODULE

The complete mathematical description of the thalamic node in our model was taken from Schellenberger Costa et al. [2016] and reads:

$$\tau_t \dot{V}_t = -J_t^{\text{leak}} - J_t^e - J_t^i - C_m^{-1}\tau_t \left(I_t^{LK} + I_t^T + I_h\right) \tag{S1}$$

$$\tau_r \dot{V}_r = -J_r^{\text{leak}} - J_r^e - J_r^i - C_m^{-1}\tau_r \left(I_r^{LK} + I_r^T\right) \tag{S2}$$

$$J_k^{\text{leak}} = (V_k - E_{\text{leak}}) \tag{S3}$$

$$J_k^e = w_e s_{ek}(V_k - E_e) \tag{S4}$$

$$J_k^i = w_i s_{ik}(V_k - E_i) \tag{S5}$$

$$I_k^{\text{LK}} = g_{LK}(V_k - E_K) \tag{S6}$$

$$I_k^T = g_k^T m_k^\infty m_k^\infty h_k(V_k - E_{Ca}) \tag{S7}$$

$$I_h = g_h(m_{h1} + g_{inc}m_{h2})(V_t - E_h) \tag{S8}$$

$$\ddot{s}_{et} = \gamma_e^2 \left(N_{ctx\to thal} \cdot r_{\text{ctx,E}} + \phi'(t) - s_{et}\right) - 2\gamma_e \dot{s}_{et} \tag{S9}$$

$$\ddot{s}_{er} = \gamma_e^2 \left(N_{rt}r_t + N_{ctx\to thal} \cdot r_{\text{ctx,E}} - s_{er}\right) - 2\gamma_e \dot{s}_{er} \tag{S10}$$

$$\ddot{s}_{it} = \gamma_i^2 \left(N_{tr}r_r - s_{it}\right) - 2\gamma_i \dot{s}_{it} \tag{S11}$$

$$\ddot{s}_{ir} = \gamma_i^2 \left(N_{rr}r_r - s_{ir}\right) - 2\gamma_i \dot{s}_{ir} \tag{S12}$$

$$\dot{h}_k = \frac{h_k^\infty - h_k}{\tau_k^h} \tag{S13}$$

$$\dot{m}_{h1} = \left(m_h^\infty(1 - m_{h2}) - m_{h1}\right)/\tau_h^m - k_3 P_h m_{h1} + k_4 m_{h2} \tag{S14}$$

$$\dot{m}_{h2} = k_3 P_h m_{h1} - k_4 m_{h2} \tag{S15}$$

$$[\dot{\text{Ca}}] = \alpha_{\text{Ca}} I_t^T - ([\text{Ca}] - [\text{Ca}_0])/\tau_{\text{Ca}} \tag{S16}$$

$$\dot{\phi}' = \frac{-\phi'}{\tau_{OU}} + \sigma_{TCR}\xi, \tag{S17}$$

with subscripts $k \in \{r, t\}$ with $r$ standing for TRN and $t$ for TCR population. Subscript $e$ ($i$) denotes excitatory (inhibitory) synaptic type. $\xi$ is drawn from random Gaussian white noise process with zero mean and unit variance. The gating functions are given by

$$m_t^\infty = \frac{1}{1 + \exp\left(-(V_t + 59)/6.2\right)} \tag{S18}$$

$$m_r^\infty = \frac{1}{1 + \exp\left(-(V_r + 52)/7.4\right)} \tag{S19}$$

$$h_t^\infty = \frac{1}{1 + \exp\left((V_t + 81)/4\right)} \tag{S20}$$

$$h_r^\infty = \frac{1}{1 + \exp\left((V_r + 80)/5\right)} \tag{S21}$$

$$\tau_t^h = \left(30.8 + (211.4 + \exp((V_t + 115.2)/5))/(1 + \exp((V_t + 86)/3.2))\right)/3^{1.2} \tag{S22}$$

$$\tau_r^h = \left(85 + 1/(\exp((V_r + 48)/4) + \exp(-(V_r + 407)/50))\right)/3^{1.2} \tag{S23}$$

$$m_h^\infty = \frac{1}{1+\exp\left((V_t+75)/5.5\right)} \tag{S24}$$

$$\tau_h^m = (20+1000/(\exp((V_t+71.5)/14.2)+\exp(-(V_t+89)/11.6))) \tag{S25}$$

$$P_h = \frac{k_1[\mathrm{Ca}]^{n_P}}{k_1[\mathrm{Ca}]^{n_P}+k_2}. \tag{S26}$$

And finally, the firing rate transfer function obeys

$$r_k = \frac{r_k^{max}}{1+\exp\left(-(V_k-\theta)/\sigma_k\right)}. \tag{S27}$$

## S7 THALAMOCORTICAL MODEL PARAMETERS.

**Table S1. Thalamic node parameters.** Taken from Schellenberger Costa et al. [2016].

| Parameter | Value | Description |
|---|---|---|
| $C_m$ | $1\ \mu\mathrm{F/cm}^2$ | membrane capacitance |
| $\tau_t$, $\tau_r$ | 20 ms | membrane time constant of TCR and TRN |
| $r_t^{\mathrm{max}}$, $r_r^{\mathrm{max}}$ | 0.4 kHz | maximal firing rate of TCR and TRN |
| $\theta$ | -58.5 mV | firing threshold (half activation) |
| $\sigma_t$, $\sigma_r$ | 6 mV | inverse neural gain |
| $\gamma_e$ | 0.07 kHz | synaptic rate constant of AMPA synapses |
| $\gamma_i$ | 0.1 kHz | synaptic rate constant of GABA synapses |
| $N_{tr}$ | 5 | connectivity constant TRN $\rightarrow$ TCR |
| $N_{rt}$ | 3 | connectivity constant TCR $\rightarrow$ TRN |
| $N_{rr}$ | 25 | recurrent connectivity constant in TRN |
| $w_e$,$w_i$ | 1 ms | synaptic weights |
| $g_t^T$ | $3\ \mathrm{mS/cm}^2$ | conductance of Ca current in TCR |
| $g_r^T$ | $2.3\ \mathrm{mS/cm}^2$ | conductance of Ca current in TRN |
| $g_h$ | [0.0–0.08] $\mathrm{mS/cm}^2$ | conductance of rectifying current in TCR |
| $g_{LK}$ | [0.0–0.08] $\mathrm{mS/cm}^2$ | conductance of K leak current in TCR and TRN |
| $E_{\mathrm{leak}}$ | -70 mV | Nernst reversal potential for leak channels |
| $E_e$ | 0 mV | Nernst reversal potential of AMPA channels |
| $E_i$ | -70 mV | Nernst reversal potential of GABA channels |
| $E_{Ca}$ | 120 mV | Nernst reversal potential of Ca channels |
| $E_K$ | -100 mV | Nernst reversal potential of K channels |
| $E_h$ | -40 mV | Nernst reversal potential of rectifying channels |
| $\alpha_{\mathrm{Ca}}$ | $-51.8\cdot10^{-6}$ mM/mAms | Ca influx rate |
| $\tau_{\mathrm{Ca}}$ | 10 ms | Ca time constant |
| $[\mathrm{Ca}_0]$ | $2.4\cdot10^{-4}$ mM | Ca resting state concentration |
| $k_1$ | $2.5\cdot10^{7}$ kHz | reaction velocities of rectifying current |
| $k_2$ | $4.0\cdot10^{-4}$ kHz | |
| $k_3$ | $1.0\cdot10^{-1}$ kHz | |
| $k_4$ | $1.0\cdot10^{-3}$ kHz | |
| $n_P$ | 4 | number of Ca binding sites |
| $g_{inc}$ | 2.0 | conductivity scaling of rectifying current |

## REFERENCES

Schellenberger Costa M, Weigenand A, Ngo HVV, Marshall L, Born J, Martinetz T, et al. A thalamocortical neural mass model of the EEG during NREM sleep and its response to auditory stimulation. *PLoS*

**Table S2. Cortical node parameters.** Taken from Cakan and Obermayer [2020].

| Parameter | Value | Description |
|---|---|---|
| $\sigma^{\text{ext}}$ | $1.5\ \text{mV}/\sqrt{\text{ms}}$ | standard deviation of external input |
| $K_E$ | 800 | number of excitatory inputs per neuron |
| $K_I$ | 200 | number of inhibitory inputs per neuron |
| $K_{E,\text{global}}$ | 250 | number of excitatory inputs from each global area |
| $c_{EE}$, $c_{IE}$ | $0.3\ \text{mV}/\text{ms}$ | maximum AMPA PSC amplitude |
| $c_{II}$, $c_{EI}$ | $0.5\ \text{mV}/\text{ms}$ | maximum GABA PSC amplitude |
| $c_{\text{global}}$ | $0.4\ \text{mV}/\text{ms}$ | postsynaptic PSC amplitude for global connectome |
| $J_{EE}$ | $2.43\ \text{mV}/\text{ms}$ | maximum synaptic current E→E |
| $J_{IE}$ | $2.6\ \text{mV}/\text{ms}$ | maximum synaptic current E→I |
| $J_{EI}$ | $-3.3\ \text{mV}/\text{ms}$ | maximum synaptic current I→E |
| $J_{EE}$ | $-1.64\ \text{mV}/\text{ms}$ | maximum synaptic current I→I |
| $\tau_{\text{s},E}$ | 2 ms | excitatory synaptic time constant |
| $\tau_{\text{s},I}$ | 5 ms | inhibitory synaptic time constant |
| $d_E$ | 4 ms | synaptic delay to excitatory neurons |
| $d_I$ | 2 ms | synaptic delay to inhibitory neurons |
| $\tau_m$ | $C/g_L = 20$ ms | membrane time constant |
| $a$ | 0 nS | subthreshold adaptation conductance |
| $b$ | 15 pA | spike-triggered adaptation increment |
| $E_A$ | -80 mV | Nernst reversal potential for adaptation current |
| $\tau_A$ | 1000 ms | adaptation current time constant |

**Table S3. Connected model parameters.** Almost all parameters here were subject to change during our investigation. This table contains the typical values of respective parameters. Different parameter values than the ones written here are mentioned in the text and the respective figures captions. In the case of parameters which change dynamical properties of the model, we indicate the range of possible values.

| Parameter | Typical value | Description |
|---|---|---|
| $N_{ctx \to thal}$ | 1.2 | connectivity strength cortex → thalamus |
| $N_{thal \to ctx}$ | 0.12 | connectivity strength thalamus → cortex |
| $d_{ctx,thal}$ | 13 ms | synaptic thalamocortical delay |
| $\tau_{OU}$ | 5 ms | time-scale of the Ornstein–Uhlenbeck process |
| $\sigma_E$ | $[0.0\text{–}0.05]\ \text{mV}/\text{ms}^{3/2}$ | noise variance for the cortical E population |
| $\sigma_I$ | $[0.0\text{–}0.05]\ \text{mV}/\text{ms}^{3/2}$ | noise variance for the cortical I population |
| $\sigma_{TCR}$ | $[0.0\text{–}0.005]\ \text{mV}/\text{ms}^{3/2}$ | noise variance for the thalamic TCR population |
| $\mu_E$ | $[0.0\text{–}5.0]\ \text{mV}/\text{ms}$ | mean drift of the Ornstein–Uhlenbeck process for cortical E population |
| $\mu_I$ | $[0.0\text{–}5.0]\ \text{mV}/\text{ms}$ | mean drift of the Ornstein–Uhlenbeck process for cortical I population |
| $\mu_{TCR}$ | $0\ \text{mV}/\text{ms}$ | mean drift of the Ornstein–Uhlenbeck process for thalamic TCR populatio |
| $\xi$ | $\sim \mathcal{N}(0,1)$ | Gaussian white noise process |

*Computational Biology* **12** (2016) e1005022.

Cakan C, Obermayer K. Biophysically grounded mean-field models of neural populations under electrical stimulation. *PLoS Computational Biology* **16** (2020) e1007822.
